# Biocatalytic synthesis of acylated derivatives of troxerutin: their bioavailability and antioxidant properties in vitro

**DOI:** 10.1186/s12934-018-0976-x

**Published:** 2018-08-22

**Authors:** Xuan Xin, Mengmeng Zhang, Xiaofeng Li, Furao Lai, Guanglei Zhao

**Affiliations:** 1 School of Food Science and Engineering, South China University of Technology, Wushan Road 381, Guangzhou, 510640 Guangdong China; 2 State Key Laboratory of Pulp and Paper Engineering, South China University of Technology, Wushan Road 381, Guangzhou, 510640 Guangdong China

**Keywords:** Troxerutin, Propionyl derivatives, Whole-cell biocatalysis, Bioavailability, Antioxidant activity

## Abstract

**Background:**

Flavonoid glycosides have many beneficial effects on health, but these bioactivities tend to decrease after oral administration owing to their poor lipophilicity. In this study, a facile whole-cell-based method was developed for selective preparation of monoester or diester of troxerutin, a flavonoid derivative. In addition, the bioavailabilities and antioxidant properties of troxerutin and its acylated derivatives were also investigated in cells.

**Results:**

*Pseudomonas aeruginosa* and *Pseudomonas stutzeri* cells showed high catalytic efficiency (substrate conversion > 90%) and different preferences for troxerutin, resulting in the production of its monoester (TME) and diester (TDE), respectively. The log*P* values of troxerutin, TME, and TDE were − 2.04 ± 0.10, − 0.75 ± 0.08, and 1.51 ± 0.05 and their *P*_app_ values were 0.34 × 10^−6^ ± 0.05, 0.99 × 10^−6^ ± 0.12, and 1.54 × 10^−6^ ± 0.17 cm/s, respectively. The results of hydroxyl radical, ABTS, and ORAC assays indicated that the antiradical activities of acylated derivatives did not exceed that of troxerutin, but showed higher inhibition effects upon 2,2′-azobis(2-amidinopropane) dihydrochloride-induced erythrocyte hemolysis than that of troxerutin (*P* < 0.05).

**Conclusion:**

A facile and efficient whole-cell biocatalysis method was developed to synthesize troxerutin-acylated derivatives, markedly enhancing the bioavailability and antioxidant activities of troxerutin in cells. Additionally, the mechanism underlying the observed difference in the antioxidant activities of troxerutin and its esters was ascribed to both their free radical scavenging abilities and distribution on the cell membrane surface.
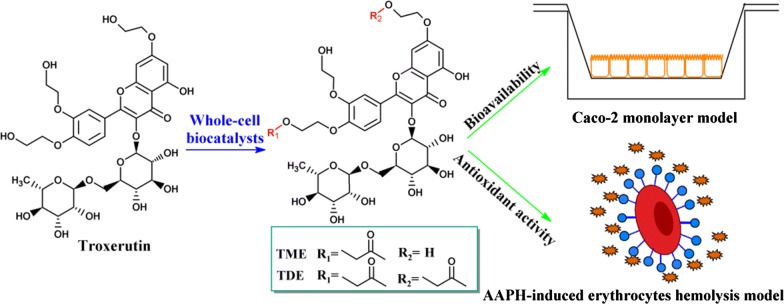

**Electronic supplementary material:**

The online version of this article (10.1186/s12934-018-0976-x) contains supplementary material, which is available to authorized users.

## Background

Flavonoids are ubiquitously present natural compounds, that have been demonstrated to have anti-thrombotic, nephro-protective, and hepato-protective effects, and thus, are widely used in food, pharmaceutical, and cosmetic products [1, 2]. As an important flavonoid, troxerutin is a tri(hydroxyethyl) derivative of rutin with multiple nutritional and pharmacological properties. However, the application of flavonoid glycosides is greatly restricted because of their poor cell membrane penetration, resulting in low oral bioavailability [3, 4]. However, this limitation may be overcome using the strategy applied for improving their lipophilicity—selectively acylation modification of flavonoids [5]. To date, acylated derivatives of flavonoids are prepared by either chemical synthesis or biocatalysis, while biocatalysis is typically carried out using free or immobilized enzymes as well as whole microbial cells [5–7]. Generally, whole-cell biocatalysts are preferable for acylation of flavonoids because they are not only more eco-friendly, show higher selectivity, and use more mild reaction conditions than chemical catalysts do, but also do not require enzyme purification, have low production costs, and exhibit less enzyme inactivation compared to the free enzyme [8, 9]. Whole-cell biocatalysis shows promise for industrial application to supplement or replace chemical or enzymatic synthesis for flavonoid acylated derivatives. Previously, chemical and enzyme catalysis have been used to synthesize troxerutin esters [6, 10]. However, acylation of troxerutin by whole-cell biocatalysis has not been reported.

According to the biopharmaceutics classification system (BCS), only compounds with proper lipophilicity can improve their absorption across the gastrointestinal tract [11]. However, few studies have investigated the intestinal permeability of flavonoid-acylated derivatives. Caco-2 cells separated from a human colorectal adenocarcinoma cell line have been widely applied to study the absorption, metabolism, and transport of compounds because of their similar microvilli structures as the small intestine and because they express some enzymes similar to intestinal epithelial cells [12]. Importantly, the extent of compounds permeability across Caco-2 monolayers is closely related to their absorption through the gastrointestinal tract [13]. These factors can be used to predict the bioavailability of flavonoid-acylated derivatives. No previous studies have examined the absorption of troxerutin and its acylated derivatives. Similarly, fatty acyl-modification of flavonoids also affects their bioactivities, particularly the ability of scavenging radicals [14, 15]. This fact is more critical for the position of substitutions during the acylation reaction as the phenolic hydroxyl is the main contributor to free radical scavenging. However, there was only one report of the free radical scavenging capacity of troxerutin esters, in which antioxidant activity was tested by a simple chemical method [6], and there have been no reports of the antioxidant activity of troxerutin ester in cells. The antioxidant activity in cells and chemical experiments were reported to greatly differ [16].

**Scheme 1:**
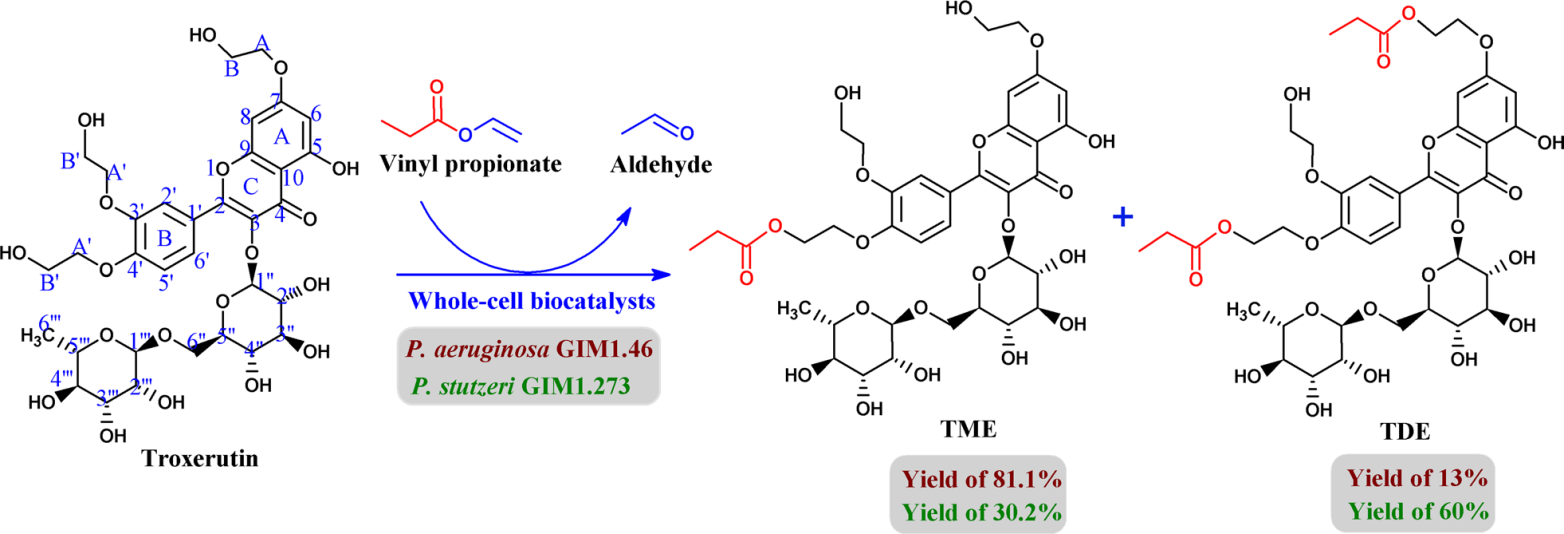
Acylation of troxerutin with vinyl propionate catalyzed by *P. aeruginosa* and *P. stutzeri* cells

In our previous study, we screened whole-cell biocatalysts from various microbial sources. Whole cells of *Pseudomonas aeruginosa* GIM 1.46 and *P. stutzeri* GIM 1.273 were capable of synthesizing troxerutin-acylated derivatives [17]. In the present study, we developed a new whole-cell biocatalytic method for producing mono- or di-esters of troxerutin using the two *Pseudomonas* strains (Scheme 1). The bioavailabilities of acylated derivatives of troxerutin were also evaluated. In addition, the antioxidant activities of troxerutin and its acylated derivatives were investigated by radical scavenging assays, oxygen radical absorbance capacity (ORAC) assay, and 2,2′-azobis(2-amidinopropane) dihydrochloride (AAPH)-induced erythrocyte hemolysis.

## Methods

### Microorganisms and materials

*Pseudomonas stutzeri* GIM 1.273 and *P. aeruginosa* GIM 1.46 were purchased from Guangdong Institute of Microbiology (Guangzhou, Guangdong, China). Troxerutin (97% purity), AAPH, ABTS, fluorescein and 6-hydroxy-2,5,7,8-tetramethylchroman-2-carboxylic acid (Trolox) were purchased from Aladdin (Los Angeles, CA, USA). Vinyl propionate (VP, 98% purity) was purchased from TCI (Tokyo, Japan). Phosphate-buffered saline (PBS, pH 7.4), Hank’s balanced salt solution (HBSS), fetal bovine serum (FBS), Dulbecco’s modified Eagle’s medium (DMEM), streptomycin, and penicillin were purchased from Gibco Life Technologies (Grand Island, NY, USA). The microscale malondialdehyde (MDA) and alkaline phosphatase (ALP) assay kits were from Beyotime Institute of Biotechnology (Shanghai, China). Caco-2 cells were obtained from the Medical College of Sun Yat-Sen University (Guangzhou, China). Soybean oil was purchased locally. All other chemicals were obtained from commercial sources and were of the highest available purity.

### Preparation of whole-cell biocatalysts

*Pseudomonas stutzeri* GIM 1.273 and *P. aeruginosa* GIM 1.46 cells were cultivated as described previously [18]. The cells were collected by centrifugation and the liquid supematant containing culture media and extracellular enzymes were removed. Then the cells were washed twice with distilled water to remove the residual medium from the cell surfaces. The washed pellets were freeze-dried by vacuum, ground into powder as whole-cell biocatalysis, and stored at 4 °C.

### General procedure for whole-cell-mediated acylation of troxerutin

In a typical experiment, 1 mL 35% *n*-heptane/pyridine (v/v) binary solvents containing 30 mM troxerutin, 900 mM VP, and 50 mg/mL whole-cell catalysts were added to a 5-mL Erlenmeyer flask (rubber serum cap) and incubated by shaking at 180 rpm and 40 °C for 120 h. Aliquots (20 µL) were removed at specified time intervals from the reaction mixture, diluted 50 times with methanol, and analyzed by high-performance liquid chromatography (HPLC). Each reaction was conducted in triplicate. The control experiments clearly showed that the acylation reaction does not proceed in the absence of cell biocatalysts. For the structural identification of products and their determination of log*P*, *P*_app_, and antioxidant activities, the reaction mixtures with scaling up were centrifuged to remove cell pellets. The reaction liquids were purified with methanol via reduced pressure distillation and then were isolated to different component with thin-layer chromatography (TLC) method (eluent, ethyl acetate/methanol/H_2_O, 15:3.6:0.5, v/v/v). After crystallization under vacuum drying, two products were obtained as brown powders.

### Determination of 1-octanol/water partition coefficient

The 1-octanol/water partition coefficients of troxerutin and its propionyl derivatives were measured as previously described [19]. Each compound was fully mixed with 1-octanol and then vigorously mixed with an equal amount of water. After equilibration for 30 min, the concentrations of compound in each layer were respectively measured by HPLC. The results are shown as common logarithms (log*P*).

### Transport experiment in Caco-2 monolayers

The absorptions of troxerutin and its acylated derivatives was measured as described by Chen et al. [12]. Caco-2 cells were cultured in DMEM supplemented with 10% FBS, 100 μg/mL streptomycin, and 100 U/mL penicillin at 37 °C in an atmosphere of 5% CO_2_ and the medium was changed every 2 days. Caco-2 cells used in this study were seeded onto 12-well translucent Transwell inserts (0.4 μm pore size, Corning Costar, Corning, NY, USA) at a density of 2.5 × 10^5^ cells/mL and grown for 3 weeks. Transepithelial electrical resistance (TEER) and lucifer yellow were measured to detect the integrity of Caco-2 monolayers. TEER values were measured by using a voltammeter (Millicell ERS, Millipore, Bedford, MA, USA) and the amount of lucifer yellow transported to the basolateral side in a 2.5-h period was quantified by using a fluorospectrometer (Fluoroskan Ascent FL, Thermo Labsystems, Helsinki, Finland) at an excitation wavelength of 425 nm and emission wavelength of 530 nm. ALP activity was measured to detect the differentiation degree of Caco-2 monolayers by using an ALP Assay Kit.

Samples (100 μM, 0.5 mL) were placed on the apical (AP) side along with 1.5 mL of HBSS added to the basolateral (BL) side. Propranolol and furosemide were used as controls. The volume removed from the recipient side was replaced with fresh HBSS at 30, 60, 90, 120, and 150 min. The concentrations on the BL side were measured by HPLC. The apparent permeability coefficients (*P*_app_, cm/s) were calculated according to the equation:

$$P_{\text{app}} = \left( {{{{\text{d}}Q} \mathord{\left/ {\vphantom {{{\text{d}}Q} {{\text{d}}t}}} \right. \kern-0pt} {{\text{d}}t}}} \right) \times {V \mathord{\left/ {\vphantom {V {\left( {A \times C_{0} } \right)}}} \right. \kern-0pt} {\left( {A \times C_{0} } \right)}},$$

where the d*Q*/d*t* is the permeability rate (μM/s) calculated from the slope of the amount of tested compounds transported versus time, *V* is the volume of the basolateral chamber (mL), *A* is the surface area of the inset (cm^2^), and *C*_0_ is the initial concentration (μM) of the samples.

### HPLC analysis

The reaction mixture was analyzed by reversed-phase HPLC on a 4.6 mm × 250 mm (5 µm) Zorbax SB-C18 column (Agilent Technologies Co., Ltd., Santa Clara, CA, USA) using a Waters 600E pump and Waters 2996UV/photodiode array detector (Milford, MA, USA) at 350 nm. A mixture of methanol and water (62/38, v/v) with glacial acetic acid (0.1%, v/v) was used as the mobile phase at a flow rate of 0.9 mL/min. The retention time for troxerutin, troxerutin monoester (TME), and troxerutin diester (TDE) were 3.6, 5.2, and 7.3 min, respectively. The conversion of troxerutin, yield of TME or TDE, initial rate, and regioselectivity of the reaction were calculated using the equations shown below, respectively:

$${\text{Conversion}}\;\left( \% \right) = {{\left( {{\text{A}}_{\text{t}} - {\text{A}}_{ 0} } \right)} \mathord{\left/ {\vphantom {{\left( {{\text{A}}_{\text{t}} - {\text{A}}_{ 0} } \right)} {{\text{A}}_{ 0} \times 100}}} \right. \kern-0pt} {{\text{A}}_{ 0} \times 100}}$$

$${\text{Yield}}\;\left(\% \right) = {{{\text{P}}_{t} } \mathord{\left/ {\vphantom {{{\text{P}}_{t} } {{\text{S}}_{ 0} }}} \right. \kern-0pt} {{\text{S}}_{ 0} }} \times 100$$

$${\text{V}}_{0} \; \left( {{{\text{mmol}} \mathord{\left/ {\vphantom {{\text{mmol}} {{\text{L}}\;{\text{h}}}}} \right. \kern-0pt} {{\text{L}}\;{\text{h}}}}} \right) = {{\left( {{\text{A}}_{\text{t}} - {\text{A}}_{0} } \right)} \mathord{\left/ {\vphantom {{\left( {{\text{A}}_{\text{t}} - {\text{A}}_{0} } \right)} {{\text{t}} \times 100}}} \right. \kern-0pt} {{\text{t}} \times 100}}$$

$${\text{Regioselectivity}}\;\left( \% \right) = {{{\text{A}}_{\text{Pi}} } \mathord{\left/ {\vphantom {{{\text{A}}_{\text{Pi}} } {{\text{A}}_{\text{total}} }}} \right. \kern-0pt} {{\text{A}}_{\text{total}} }} \times 100$$

where the A_t_ is the peak area of troxerutin after the reaction, A_0_ is the peak area of troxerutin before the reaction, P_t_ is the molarity of TME or TDE after the reaction (mM), S_0_ is the initial molarity of troxerutin (mM), t is the reaction time (h), A_Pi_ is the peak area of the target product (pi); and A_total_ is the sum of the peak area of all products. The average error for this assay was less than 1.0%.

### Structural characterization of the products

The products were structurally characterized by HPLC, mass spectra, FT-IR spectra, and ^13^C-nuclear magnetic resonance (NMR) (Additional file 1: Figures S1, S2, S3, and S4, respectively).

### Assay of antioxidant activity in chemical experiments

Scavenging abilities of hydroxyl and ABTS radicals, and ORAC were measured as described previously with some modifications [20–22]. A 759S UV/Vis spectrophotometer (Lengguang Technology, Shanghai, China) was used to determine the absorbance of HO· at 532 nm and ABTS^·+^ at 734 nm. ORAC analysis was conducted using a Fluoroskan Ascent microplate reader (Thermo Electron Corp., Waltham, MA, USA) at excitation and emission wavelengths of 485 and 530 nm, respectively. Trolox or vitamin C (VC) was used as a positive control, the sample concentration was 20 μM, and all experiments were repeated independently in triplicate.

### Assay for erythrocyte hemolysis induced by AAPH

The hemolysis study was performed according to the method of Zhang et al. [16] with some modifications. Erythrocytes from healthy sheep were centrifuged at 770×*g* for 10 min at 4 °C and washed three times with PBS. Equal amounts of erythrocyte suspension (20%, v/v) and sample solution (20 μM) were mixed and the mixture was incubated for 20 min at 37 °C. After adding 200 mM AAPH, incubation was continued for a further 2.5 h at the same temperature. The final reaction system was diluted with 8 mL PBS and then centrifuged at 1200×*g* for 10 min at 4 °C. The degree of hemolysis in the supernatant was determined by measuring the absorbance at 540 nm. To obtain the complete hemolysate, ultrapure water was added to the mixture. PBS was used as a negative control and Trolox was used as a positive control. These values were measured using a 756S UV/Vis spectrophotometer (Lengguang Technology). The treated supernatant was collected to measure the level of MDA. All experiments were performed independently in triplicate. The hemolysis rate was calculated using the equation:

$${\text{Hemolysis rate}}\left( \% \right) = \left( {{{{\text{OD}}_{540} \;{\text{of sample}}} \mathord{\left/ {\vphantom {{{\text{OD}}_{540} \;{\text{of sample}}} {{\text{OD}}_{540} \;{\text{of complete hemolysis}}}}} \right. \kern-0pt} {{\text{OD}}_{540} \;{\text{of complete hemolysis}}}}} \right) \times 100\% .$$

### Statistical analysis

The data were expressed as the mean ± standard deviation (SD) of three replicates. Significant differences between the means of parameters were calculated by Duncan’s multiple-range test using SPSS 17.0 software (SPSS, Inc., Chicago, IL, USA). Values of *P* < 0.05 were considered statistically significant.

## Results and discussion

### Comparison of catalytic performance of the two *Pseudomonas* strains for synthesizing troxerutin propionyl derivatives

**Fig. 1:**
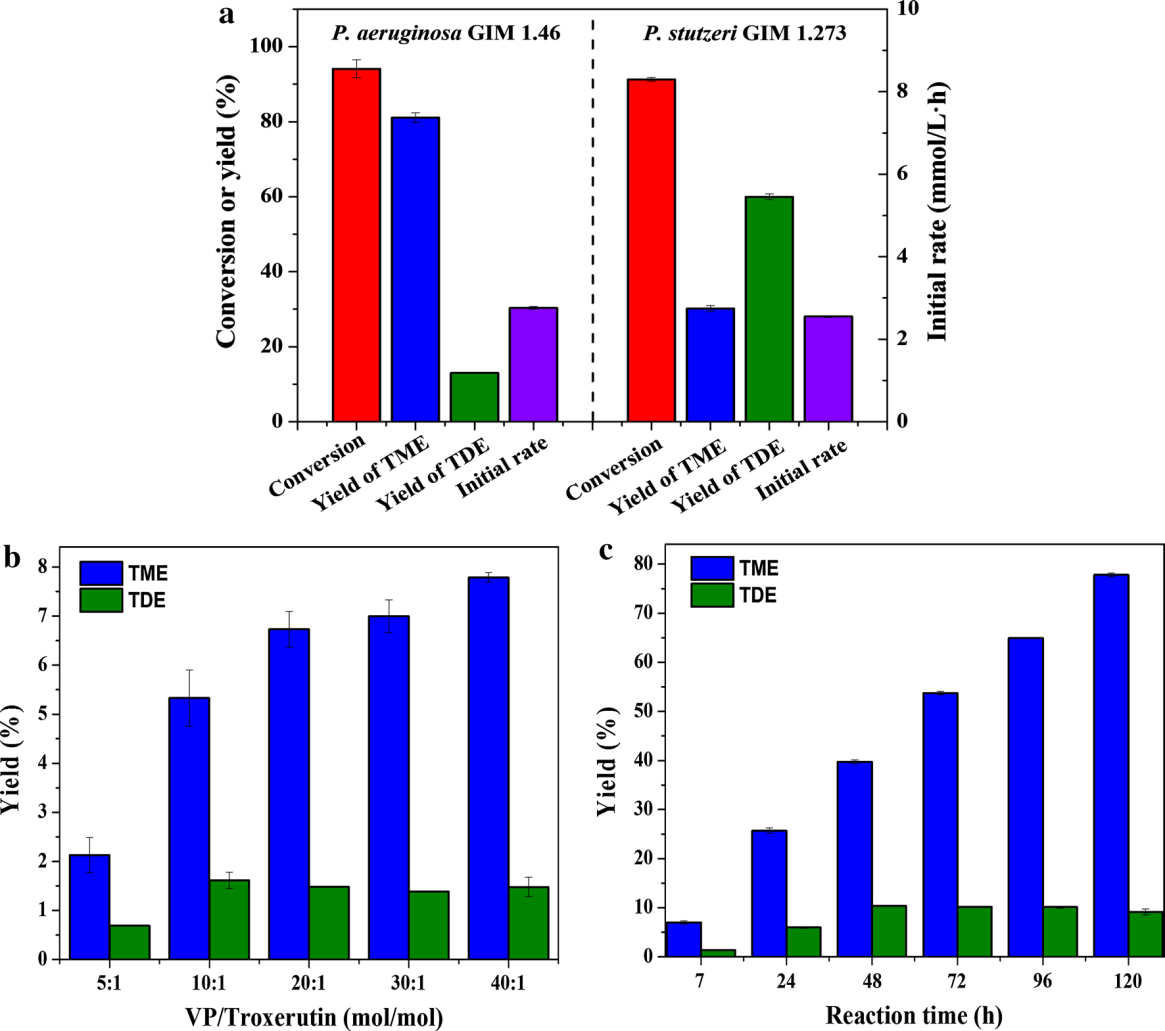
Whole-cell biocatalyst-mediated synthesis of troxerutin propionyl derivatives: **a** catalytic performances of *P. aeruginosa* cells (left of dotted line) and *P. stutzeri* cells (right of dotted line), **b** effect of molar ratio of VP/troxerutin on the catalytic activity of *P. aeruginosa* after reacting for 7 h, and **c** effect of reaction time on the catalytic activity of *P. aeruginosa* when the molar ratio of VP/troxerutin was 30 after reacting for 120 h. Experiments were performed in triplicate, and data are presented as the mean ± SD

In our previous study, whole cells of *P. aeruginosa* GIM 1.46 and *P. stutzeri* GIM 1.273 were found to be capable of catalyzing acylation of troxerutin, giving two products (the monoester TME and diester TDE). Figure 1a shows that two types of *Pseudomonas* whole cells had similar catalytic activities and efficiencies considering their similar initial rates (2.55 and 2.76 mmol/L h) and substrate conversions (94.1% and 91.3%) in the acylation of troxerutin under the optimal reaction conditions [17]. These values were much higher than those in previous reports using chemical (substrate conversion of 57%) [6] or enzymatic methods (ester yield of 59.2%) [10]. The products were characterized by electrospray ionization (ESI)-mass spectroscopy (MS), Fourier transform infrared spectroscopy (FT-IR), and ^13^C-NMR (Additional file 1: Figures S1–S4). Comparison of the FT-IR spectra of compounds showed the significant increase of carbonyl absorption intensity of the P_1_ (1726.23 cm^−1^) and the P_2_ (1727.20 cm^−1^) than that of troxerutin (1655.83 cm^−1^). Electrospray ionization (ESI)-mass spectroscopy (MS) analysis allowed the identification of P_1_ as a monoester [(M + Na)^+^, *m*/*z* 821.2483] and P_2_ as a diester [(M + Na)^+^, *m*/*z* 877.2733]. To confirm the acylation site, ^13^C-NMR spectra showed that the mono-acylation on the C_B_′-OH of B ring led to an upfield shift of the C_B_′ signal of − 2.8 ppm and a downfield shift of the C_A_′ signal of 3.49 ppm. And the di-acylation on the C_B_-OH of A ring and C_B_′-OH of B ring led to upfield shifts of C_B_′ and C_B_ (− 2.8 ppm and − 2.78 ppm, respectively) and downfield shifts of the C_A_′ and C_A_ (3.5 ppm and 3.54 ppm, respectively). Interestingly, the two types of whole cells had very different selectivities in catalyzing the acylation of hydroxyl groups of troxerutin. *P. aeruginosa* GIM 1.46 cells showed a preference for acylation at C_B_′-OH of the B ring of troxerutin, resulting in a high TME yield of 81.1%. *P. aeruginosa* GIM 1.46 cells showed difficulty in further catalyzing the acylation of other hydroxyl groups of troxerutin. Thus, the yield of diester TDE was only 13.0%. In contrast, *P. stutzeri* GIM1.273 cells showed less regioselectivity towards C_B_′-OH of the B ring of troxerutin, which can easily catalyze the acylation of both the C_B_′-OH of the B ring and C_B_-OH groups of the A ring, thus giving TDE as the dominant product (yield of 60%). Previous studies reported similar phenomena in the synthesis of other esters, which was ascribed to the excessive use of acyl donors [23, 24], as increased acyl donor concentrations significantly promoted the formation of diesters or even triesters. To explain this result, the effects of the VP/troxerutin molar ratio on monoester and diester yields by *P. aeruginosa* cells were investigated (Fig. 1b, c). The results showed that the monoester and diester were already produced during the initial stage of the reaction (7 h) with low VP/troxerutin molar ratios. No significant increase in diester yield was found when the VP/troxerutin molar ratio was increased from 5:1 to 40:1 or when the reaction time was extended from 7 to 120 h (Fig. 1b, c). Although rather high concentrations of the monoester were reached, the conversion of monoesters to diesters was not evidently enhanced. The results of these tests further indicated that the regioselectivity of this reaction was determined by the whole-cell biocatalyst rather than the molar ratio of the two substrates. We predicted that different types of cell-bound lipases exist in *P. aeruginosa* cells, resulting in different catalytic behaviors. Some of the enzymes are responsible for catalyzing the formation of monoesters, and others are used to generate diesters.

Troxerutin is a synthetic derivative of rutin and their structures are also similar, consisting of 2-phenyl-chromone and rhamnose. Biely et al. [25] synthesized the propionates of rutin using acetyl esterase of *Trichoderma reesei* as biocatalysts, giving monoester yield of 30%. The yield of rutin monoester was significantly lower than that of troxerutin monoester; this might be related to the absence of the primary hydroxyl group in rutin. However, in our previous study, rutin was also relatively good substrate for *P. aeruginosa* GIM 1.46 cells to synthesize 4‴-O-propionyl rutin with yield of 61.5% in the same reaction conditions [17], showing the *P. aeruginosa* cells have high catalytic activity and regioselectivity.

### Log*P* values and permeability of troxerutin and its propionyl derivatives

The 1-octanol/water partition coefficient (log*P*), an important pharmacokinetic parameter, is commonly used to evaluate the lipophilicity of a molecule [19]. Higher the log*P* value, higher is the lipophilicity and vice versa. As expected, acylation of troxerutin with vinyl esters resulted in an increase in its lipophilicity, as log*P* values of TME (− 0.75) and TDE (1.51) higher than their parent troxerutin (− 2.04) were observed (Table 1). Because troxerutin can also be synthesized by hydroxyethylation of rutin to improve its water solubility [26]. To confirm the improvements in their characteristics, the log*P* value of rutin was also determined. The results showed that the log*P* value of rutin (− 0.58) is higher than that of troxerutin and TME, but lower than that of TDE, further indicating that the lipophilicity of troxerutin is very poor and TDE has better lipophilicity than rutin and TME. Additionally, the log*P* value may be used to eliminate compounds that are unlikely to be easily absorbed from the intestine according to a predetermined range [19]. Compounds with log*P* values of 0–3 may have relatively good potential for absorption via oral administration [27]. Therefore, the absorption of TME and TDE may be better than that of troxerutin, but TME may not be easily absorbed through the small intestine because of its lower log*P* value than that of rutin.

**Table 1:** Log*P*, *P*_app_ and UR values for troxerutin and its propionyl derivatives in Caco-2 cell monolayers

Compound	Log*P*	*P*_app_ AP–BL (×10^−6^ cm/s)	*P*_app_ BL–AP (×10^−6^ cm/s)	UR^1^
Troxerutin	− 2.04 ± 0.10	0.34 ± 0.05 d	0.35 ± 0.03 d	0.97 ± 0.07
TME	− 0.75 ± 0.08	0.99 ± 0.12 c	1.05 ± 0.12 c	0.94 ± 0.18
TDE	1.51 ± 0.05	1.54 ± 0.17 b	1.53 ± 0.21 b	1.00 ± 0.09
Rutin	− 0.58 ± 0.13	0.06 ± 0.01 e	0.06 ± 0.02 e	1.00 ± 0.05
Propranolol	–^2^	48.90 ± 3.30 a	44.05 ± 2.98 a	1.11 ± 4.90
Furosemide	–^2^	0.85 ± 0.15 c	0.86 ± 0.11 c	0.99 ± 0.13

Experiments were performed in triplicate, and data are presented as the mean ± SD. Values with different letters (a, b, c, d, e) in an assay are significantly different one from another (*P* < 0.05) in multiple-range analysis

^1^UR =* P*_app_ AP–BL/*P*_app_ BL–AP

^2^Not measured

It should be pointed out that the absorption of flavonoids via oral administration is a very complex process and their degree of absorption depends not only on the lipophilicity of molecules, but also on the effect of transporters and enzymes on the membrane surface [28]. Thus, bioavailability prediction cannot only depend on the log*P* value. The Caco-2 monolayer has been well-recognized in studies on intestinal transport of xenobiotics [29], and thus was selected as model to further evaluate the bioavailability of troxerutin and its propionyl derivatives; the results were compared to those of the control group (rutin, propranolol, and furosemide). As demonstrated in Additional file 1: Table S1, all TEER values were higher than 400 Ω cm^2^ and showed little change before and after the experiments (*P* > 0.05). Less than 10% of lucifer yellow was recovered from the basolateral (BL) side. ALP activity on the apical (AP) side first increased and then gradually decreased over incubation time, while ALP activity on the BL side was nearly unchanged (Additional file 1: Figure S4), confirming that the formed Caco-2 monolayer, retained good integrity, and could be used in the transport experiment. Additionally, the exposure times and concentrations of all compounds used were appropriate for this experiment. Table 1 shows that the apparent permeability coefficients (*P*_app_, cm/s) of AP to BL transport were close to those of BL to AP transport, indicating that the efficiency of transport from the AP and BL sides were similar for each compound. The quotient of absorptive permeability and secretory permeability (*P*_app_ AP–BL/*P*_app_ BL–AP) was defined as the uptake ratio (UR). Because the absorption mode of a compound was predicted to be passive transport when the UR value was close to 1.0 [30], the determined UR values of troxerutin and its acylated derivatives (0.94–1.0) indicated that passive transport occurred for all compounds tested.

The BCS divided drugs into four distinct categories according to their permeability and solubility: high permeability-high solubility, low permeability-high solubility, high permeability-low solubility, and low permeability-low solubility. Propranolol is a high permeability-high solubility lipophilic drug that can be completely absorbed after oral consumption (bioavailability of > 90%). In contrast, furosemide is a low permeability-high solubility hydrophilic drug with a low bioavailability of 10–60% [12, 31]. Based on these two extremes for estimating bioavailability, we further confirmed that troxerutin and rutin have low bioavailability, as their *P*_app_ value was lower than that of furosemide. When mono-acylation modification for troxerutin was carried out to form TME, the *P*_app_ value of TME (0.99 × 10^−6^ cm/s) was higher than that of troxerutin (0.34 × 10^−6^ cm/s) and similar to that of furosemide (0.85 × 10^−6^ cm/s), indicating that the bioavailability of TME increased, but remained low. The TDE (*P*_app_ value of 1.54 × 10^−6^ cm/s) has a relatively good bioavailability, as compounds with *P*_app_ > 1.0 × 10^−6^ cm/s were easily absorbed in humans [13]. For rutin, although its log*P* value was higher than that of troxerutin, its *P*_app_ value (0.06 × 10^−6^ cm/s) was lower than that of troxerutin, further confirming that troxerutin esters have higher solubility and permeability than rutin. These results demonstrated that acyl-modification increased the lipophilicity of troxerutin and enhanced its bioavailability. TDE showed better absorption properties than TME.

However, the *P*_app_ value of TDE was much lower than that of propranolol (48.9 × 10^−6^ cm/s). This may be partly related to the much higher molecular weight of troxerutin (742.7) compare to the control propranolol (259.3), as the molecular weight of a compound is also a restrictive factor in its absorption [32, 33]. Although the absorption of TDE was better than that of troxerutin and TME in the Caco-2 monolayer model, these effects require further investigation.

### Free radical scavenging abilities of troxerutin and its propionyl derivatives

**Fig. 2:**
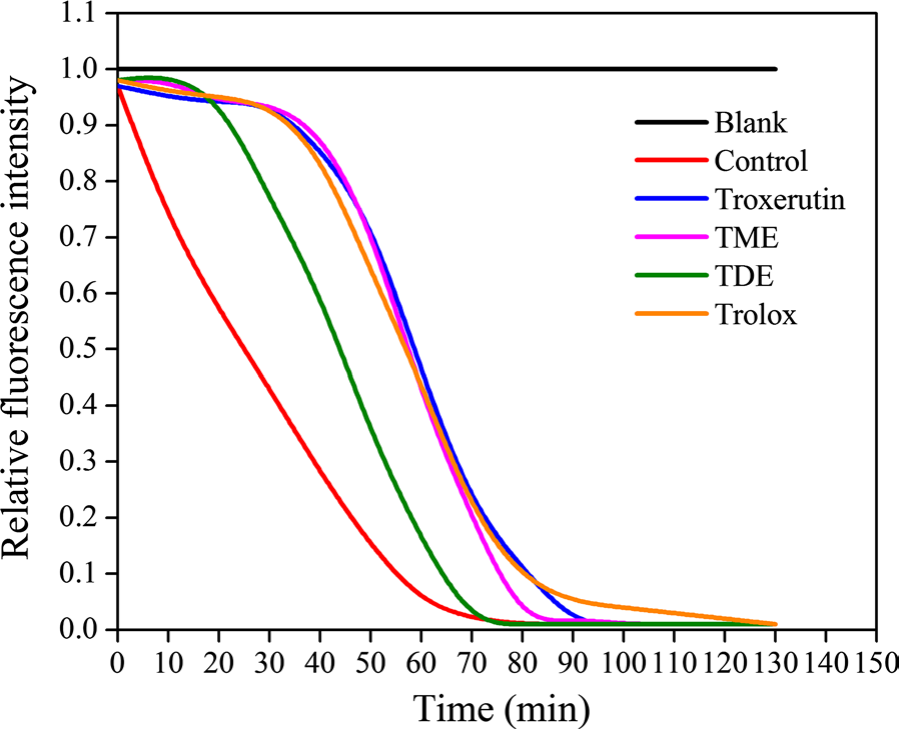
Effect of troxerutin and its propionyl derivatives on fluorescence quenching induced by AAPH. Experiments were performed in triplicate, and data are presented as the mean

Many in vivo and in vitro studies have demonstrated that the multiple bioactivities of troxerutin such as anti-inflammatory, anti-radiation, anti-apoptosis, nephro-protective, and hepato-protective properties are mainly attributed to its antioxidant activity [1, 34, 35]. To further investigate the properties of troxerutin and its derivatives as described above, their antioxidant activities were first evaluated by determining their free radical scavenging abilities (hydroxyl radicals and ABTS radicals). Table 2 shows that the scavenging abilities of all tested compounds for these two free radicals had similar trends. Troxerutin showed a relatively good ability to scavenge HO· and ABTS^·+^, as its inhibition rates for these two radicals were similar to that of the control (vitamin C). The antiradical abilities of TME and TDE were similar to or even lower than that of troxerutin. Previous studies reported that the free radical scavenging abilities of lipophilized esterified derivatives were higher than that of the parent compound, which was interpreted as the retention of phenolic hydroxyl groups on the aromatic rings [36]. Indeed, phenolic hydroxyl groups on aromatic rings were thought to be important for radical scavenging of flavonoids [37]. In this reaction, the acylation took place at the alcoholic hydroxyl groups and had minimal effect on the active site. Consequently, the free radical scavenging ability of the acylated derivatives was not expected to be lower than that of troxerutin. The ORAC assay is another traditional method for detecting the total antioxidant capacity of a compound by inhibiting the fluorescence quenching induced by AAPH. As shown in Fig. 2, the results of the ORAC assay agreed with those of the hydroxyl radicals and ABTS assay. The capacity to inhibit fluorescence quenching of all compounds tested was in this order: troxerutin ≈ TME > TDE.

**Table 2:** Free radical scavenging abilities of troxerutin and its propionyl derivatives

Compounds	Free radical scavenging ability (%)	
	HO·	ABTS^·+^
Troxerutin	97.1 ± 2.7 ab	96.8 ± 4.1 ab
TME	95.2 ± 3.5 b	90.4 ± 3.0 b
TDE	89.7 ± 1.8 c	81.5 ± 2.1 c
VC	99.3 ± 0.05 a	100 ± 0.02 a

Experiments were performed in triplicate, and data are presented as the mean ± SD. Values with different letters (a, b, c) in an assay are significantly different one from another (*P* < 0.05) in a multiple range analysis

**Fig. 3:**
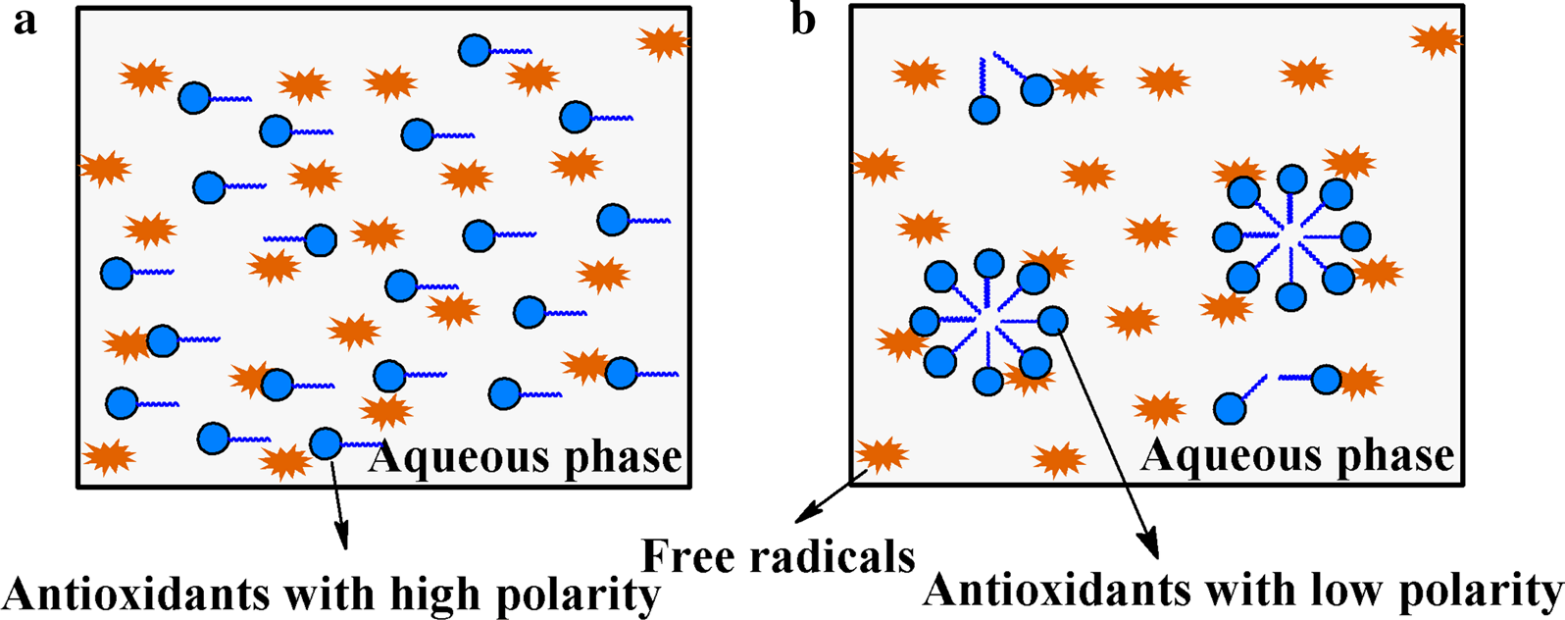
Distribution of antioxidants with different polarity in the aqueous phase of free radical scavenging experiment

Studies of the antioxidant activity of acylated flavonoids suggested that antioxidant activity depends on the reaction environment as well as lipophilic degree of acylated derivatives [36, 38]. Because aqueous reaction systems were used to determine free radical scavenging abilities, the significantly improved lipophilicity of troxerutin derivatives may influence their distribution, thus influencing their ability to scavenge free radicals. Figure 3 shows a schematic representation of the distribution of antioxidants with different polarities. At the same concentration, antioxidants with high polarity can be easily dissolved in the aqueous phase and their uniform distribution would provide sufficient contact with the free radicals, thus effectively exerting their antioxidant activities (Fig. 3a). In contrast, the distribution of the acyl derivatives of troxerutin with higher lipophilicity may be less uniform than that of troxerutin. Their lipophilic terminals would easily aggregate in the aqueous phase, resulting in lower antiradical activity (Fig. 3b). Although the lipophilicity of TME was higher than that of troxerutin, its polarity remained relatively high (log*P* < 0) and showed a better distribution than TDM, revealing the mechanism underlying the observed difference in free radical scavenging abilities of troxerutin and its esters.

### Antioxidant activities of troxerutin and its propionyl derivatives evaluated by an erythrocyte hemolysis model

**Fig. 4:**
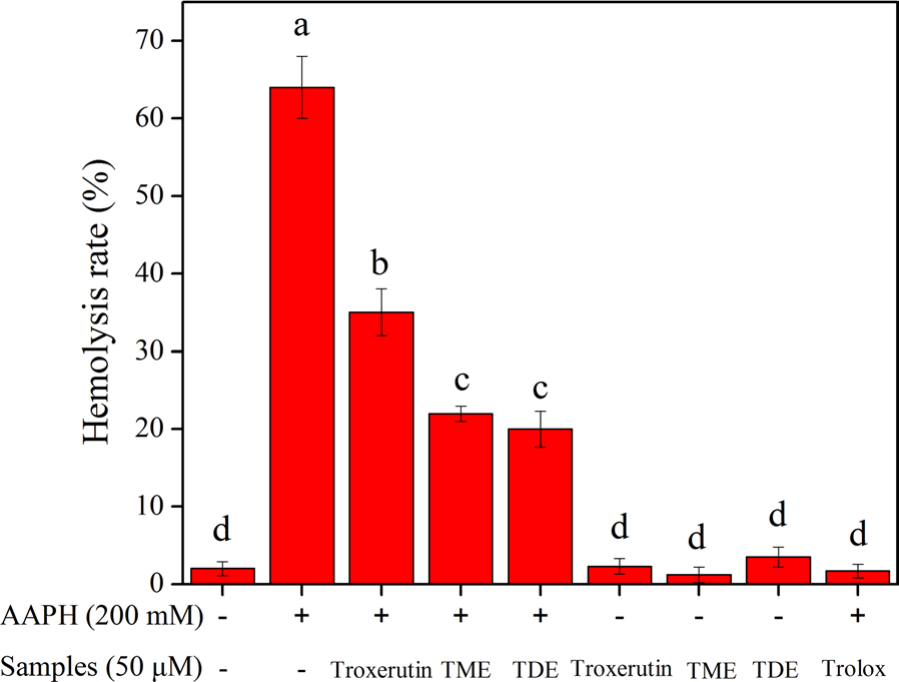
Inhibition effects of troxerutin and its propionyl derivatives on AAPH-induced erythrocyte hemolysis. Different letters indicate significant differences (*P* < 0.05) in multiple-range analysis. Experiments were performed in triplicate, and data are presented as mean ± SD

Among the various studies conducted to evaluate the antioxidant activity of a compound, an erythrocyte hemolysis model has been proposed based on the polar paradox theory in which various unsaturated fatty acids in the cell membrane of erythrocytes are susceptible to attack by AAPH-induced free radicals, leading to lipid oxidation and ultimately erythrocyte hemolysis. The presence of antioxidants may inhibit erythrocyte hemolysis by scavenging the free radicals. Additionally, esterases do not exist in the metabolic networks of erythrocytes to exclude the effect on ester hydrolysis [39]. Thus, erythrocyte hemolysis induced by the AAPH assay was used to evaluate the antioxidant activity of troxerutin and its propionyl derivatives. As shown in Fig. 4, no significant changes were observed in the hemolysis rates of all samples (control, without AAPH supplementation), while erythrocyte hemolysis was effectively inhibited by troxerutin, TME, and TDE. Their inhibition effects were in the order of troxerutin < TME ≈ TDE. The results of this ex vivo evaluation of antioxidant activities of troxerutin and its propionyl derivatives were quite different from those obtained from in vitro analysis of DPPH, ABTS, and ORAC assays.

**Fig. 5:**
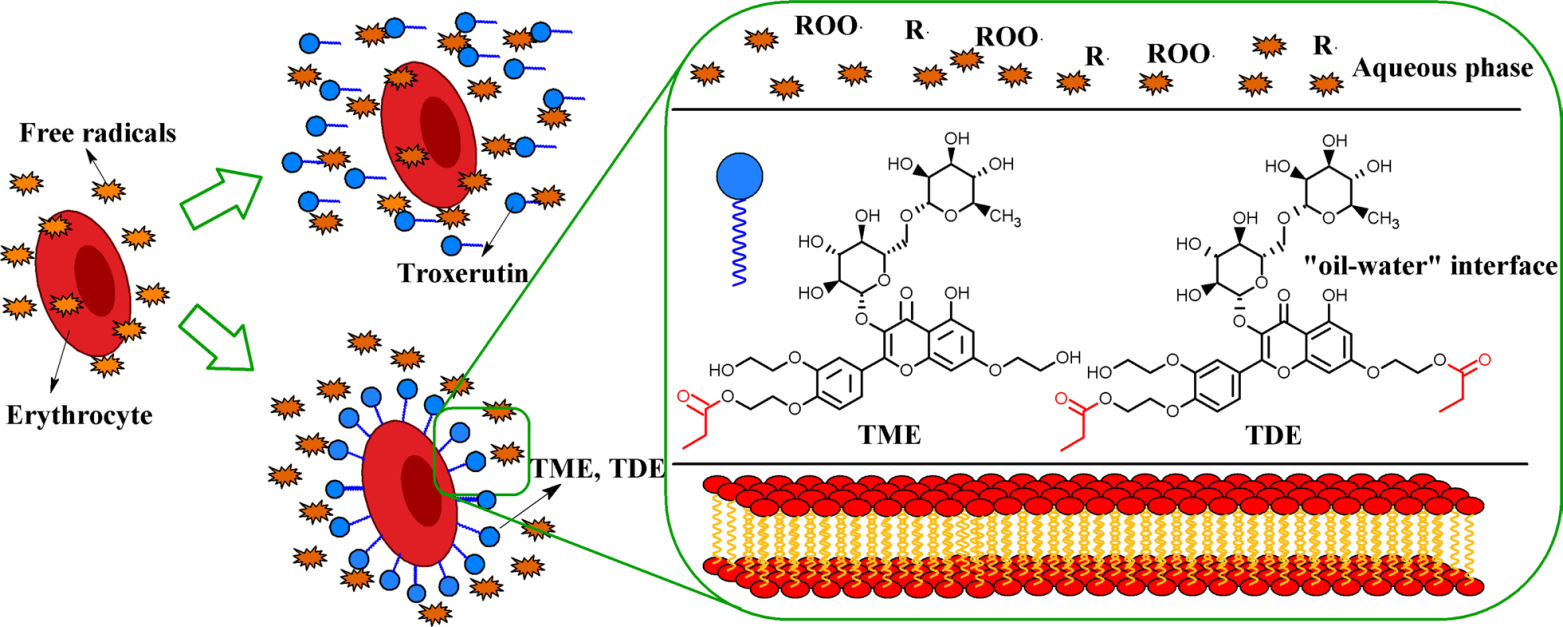
Underlying mechanism of antioxidant activities of troxerutin and its propionyl derivatives in the model of AAPH-induced erythrocyte hemolysis. Different letters in a legend indicate significant differences (*P* < 0.05) in multiple-range analysis. Experiments were performed in triplicate, and data are presented as the mean ± SD

The mechanism underlying the observed inhibition effects of troxerutin esters on erythrocyte hemolysis can be explained by the distribution of free radicals and antioxidants around the cells according to the polar paradox theory (Fig. 5). A nonpolar antioxidant is typically more effective in relatively more polar media, such as oil-in-water emulsions or liposomes [40]. The oil-in-water emulsion generally consists of three essential parts: lipid droplets, continuous aqueous phase, and oil–water interface where emulsifiers and other surface-active compounds are located. The erythrocyte is similar to a microdroplet because of its lipid bilayer, and thus, an “oil-in-water” emulsion system forms when these cells are dispersed into the aqueous phase. The cell membrane-water interaction interface is considered as a type of “oil–water” interface. In an oil-in-water emulsion, nonpolar and amphiphilic antioxidants mainly concentrate at the oil–water interface, while polar antioxidants are primarily dissolved in the aqueous phase [41, 42]. TME and TDE are amphiphilic molecules with hydrophobic groups (acyl fatty chains and parent nucleus 2-phenyl-chromone) and hydrophilic groups (aglycon and phenolic hydroxyl group), which may mainly concentrate at the “oil–water” interface to form a “protective membrane” around the cells and prevent the attack of free radicals on the cell envelope. Furthermore, the esters can protect erythrocytes from attack by free radicals by their hydrophilic terminals containing multiple active hydroxyl groups. As shown in Fig. 5, lipophilic ester molecules distribute around the erythrocyte to separate the lipid bilayer of the cells from the free radicals, and scavenge free radicals approaching erythrocytes. In contrast to esters, troxerutin is a polar compound that evenly distributes in the aqueous phase and protects erythrocytes by scavenging free radicals in the aqueous phase, but cannot separate free radicals from erythrocytes. Thus, the observed higher antioxidant activities of amphipathic ester derivatives compared to troxerutin in the AAPH-induced erythrocyte hemolysis model mainly depend on the existence of the “oil–water” interface, causing the antioxidants to form a “shield” to protect the cells.

### Effect of “oil–water” interface on membrane lipid peroxidation-inhibiting ability of troxerutin and its propionyl derivatives

**Fig. 6:**
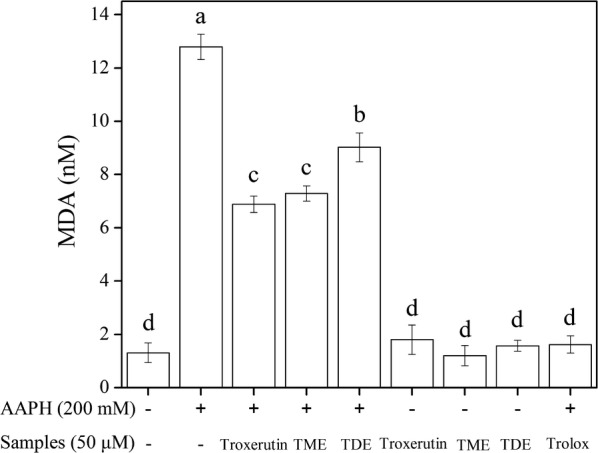
Inhibition of troxerutin and its propionyl derivatives on lipid peroxidation after complete hemolysis. Different letters in a legend indicate significant differences (*P* < 0.05) in multiple-range analysis. Experiments were performed in triplicate, and data are presented as the mean ± SD

To further demonstrate the effect of the “oil–water” interface, erythrocyte hemolysis was carried out in cold ultrapure water to completely destroy the structure of the cell membrane. The lysate was then treated with AAPH and troxerutin or its propionyl derivatives. The generation of reactive oxygen species induced by AAPH can cause lipid peroxidation of the cell membrane, leading to MDA release. Figure 6 shows the level of MDA release and inhibition ability of all samples for membrane lipid peroxidation, which was in the order of troxerutin ≈ TME > TDE; these results are consistent with those of their free radical scavenging abilities. The results indicated that when the cell membrane was destroyed, TME and TDE could not aggregate around the cells to form a “shield”. The results further confirmed that the intact cell membrane or “oil–water” interface played an important role in the antioxidant activity of amphipathic acylated derivatives.

## Conclusions

In this study, we successfully established a controllable biocatalytic method for efficient synthesis of TME by using *P. aeruginosa* cells or TDE by using *P. stutzeri* cells, with substrate conversions greater than 90%. Additionally, some promising properties of troxerutin esters were analyzed, revealing that TME and TDE had better bioavailability than troxerutin and TDE had the best absorption; the protective effects of troxerutin esters on cells under oxidative stress conditions were enhanced. The mechanism underlying the increased antioxidant activities of troxerutin esters was also determined. These findings indicated the potential for using troxerutin and its derivatives in food, cosmetics, and pharmaceutical industries and promoting the utilization of flavonoid glycosides for producing high-value bioactive products. We are currently evaluating whole-cell strategies for biocatalytic modification of other natural bioactive compounds.

## Additional file

**Additional file 1.** TEER values and the permeability of lucifer yellow transported from the AP to BL side before and after experiments; HPLC chromatograms; mass chromatograms; FT-IR spectra; ^13^C-NMR spectrum; activity of ALP in Caco-2 cells.

## Abbreviations

TME: troxerutin monoester
TDE: troxerutin diester
VP: vinyl propionate
*P. aeruginosa*: *Pseudomonas aeruginosa*
*P. stutzeri*: *Pseudomonas stutzeri*
BCS: biopharmaceutics classification system
ORAC: oxygen radical absorbance capacity
AAPH: 2,2′-azobis(2-amidinopropane) dihydrochloride
HBSS: Hank’s balanced salt solution
DMEM: Dulbecco’s modified Eagle’s medium
FBS: fetal bovine serum
ALP: alkaline phosphatase
TEER: transepithelial electrical resistance
AL: apical
BL: basolateral
