# Supplementary material for: Biocatalytic synthesis of acylated derivatives of troxerutin: their bioavailability and antioxidant properties in vitro
Source: Microb Cell Fact. 2018 Aug 22;17:130. doi: 10.1186/s12934-018-0976-x (PMC6106897; doi:10.1186/s12934-018-0976-x)
Supplement: Supplementary file 1 — Additional file 1. TEER values and the permeability of lucifer yellow transported from the AP to BL side before and after experiments; HPLC chromatograms; mass chromatograms; FT-IR spectra; 13C-NMR spectrum; activity of ALP in Caco-2 cells. [file 12934_2018_976_MOESM1_ESM.docx]

**Additional files**

**Biocatalytic synthesis of acylated derivatives of troxerutin: their bioavailability and antioxidant properties *in vitro***

**Authors:**

Xuan Xin^1^, Mengmeng Zhang^1^, Xiaofeng Li^1*^, Furao Lai^1^, Guanglei Zhao^2*^

**Corresponding authors:**

*Dr. Xiaofeng Li, Tel: (+86)20-22236819; E-mail: [xflibio@scut.edu.cn](mailto:xflibio@scut.edu.cn) ; Fax: (+86)20-87112853

*Dr. Guanglei Zhao, Tel: (+86)20-87111770; E-mail: [glzhao@scut.edu.cn](mailto:glzhao@scut.edu.cn(G.l.%20Zhao);); Fax: (+86)20-87111770

**Total No. of Pages:** 11 (Page S1-S11)

**Total No. of Tables:** 2 (Table S1-S2)

**Table No. of Figures:** 4 (Figure S1-S5)

**Table S1.** TEER values and the permeability of lucifer yellow transported from the AP to BL side before and after experiments ^a^

| Compounds | TEER (Ω·cm^2^) | | | Lucifer yellow (%) | |
| --- | --- | --- | --- | --- | --- |
|  | Before | After |  | Before | After |
| Troxerutin | 495 ± 5 a | 497 ± 4 a |  | 4.7 ± 0.6 a | 5.3 ± 0.3 a |
| TME | 502 ± 7 a | 499 ± 5 a |  | 5.0 ± 1.6 a | 4.3 ± 1.1 a |
| TDE | 484 ± 8 a | 481 ± 2 a |  | 4.4 ± 0.9 a | 3.9 ± 1.5 a |
| Propranolol | 488 ± 11 a | 485 ± 9 a |  | 4.8 ± 1.2 a | 5.1 ± 1.1 a |
| Furosemide | 491 ± 9 a | 493 ± 7 a |  | 3.9 ± 1.7 a | 4.6 ± 1.6 a |

^a^ Experiments were performed in triplicate, and data are presented as the mean ± SD. Same letter (a) showed no significant difference (*p* > 0.05) in the same compound. There are no significant differences in TEER values and the permeability of lucifer yellow from the AP to BL side before and after the experiments.

**Table S2.** The ^13^C-NMR data of Troxerutin, TME and TDE

| Carbon number | Troxerutin | TME | Chemical shift Differences (∆ppm) | TDE | Chemical shift Differences (∆ppm) |
| --- | --- | --- | --- | --- | --- |
| C-4 | 177.93 | 177.88 | 0.05 | 177.82 | 0.11 |
| C-7 | 165.11 | 165.13 | −0.02 | 165.13 | −0.02 |
| C-9 | 161.29 | 161.43 | −0.14 | 161.71 | −0.42 |
| C-5 | 157.01 | 156.97 | 0.04 | 157.01 | 0 |
| C-2 | 156.89 | 156.82 | 0.07 | 156.71 | 0.18 |
| C-4′ | 151.37 | 150.85 | 0.52 | 150.84 | 0.53 |
| C-3′ | 148.00 | 148.14 | −0.14 | 148.16 | −0.16 |
| C-3 | 134.16 | 134.31 | −0.15 | 134.41 | −0.25 |
| C-1′ | 123.03 | 123.07 | −0.04 | 123.07 | −0.04 |
| C-6′ | 122.87 | 122.88 | −0.01 | 123.04 | −0.17 |
| C-5′ | 114.99 | 115.38 | −0.39 | 115.35 | −0.36 |
| C-2′ | 113.32 | 113.92 | −0.6 | 113.90 | −0.58 |
| C-10 | 105.52 | 105.6 | −0.08 | 105.71 | −0.19 |
| C-1′′′ | 101.37 | 101.35 | 0.02 | 101.32 | 0.05 |
| C-6 | 98.83 | 98.92 | −0.09 | 98.91 | −0.08 |
| C-8 | 93.34 | 93.27 | 0.07 | 93.12 | 0.22 |
| C-3′′ | 76.87 | 76.91 | −0.04 | 76.94 | −0.07 |
| C-5′′ | 76.42 | 76.39 | 0.03 | 76.41 | 0.01 |
| C-2′′ | 74.64 | 74.58 | 0.06 | 74.61 | 0.03 |
| C-4′′′ | 72.24 | 72.25 | −0.01 | 72.29 | −0.05 |
| C-3′′′ | 71.08 | 71.18 | −0.1 | 71.18 | −0.1 |
| C-2′′′ | 71.08 | 71.13 | −0.05 | 71.18 | −0.1 |
| C-4′′ | 70.93 | 70.93 | 0 | 70.88 | 0.05 |
| C-A′ | 70.83 | 67.34 | **3.49** | 67.33 | **3.5** |
| C-A′ | 70.79 | 70.73 | 0.06 | 70.74 | 0.05 |
| C-A | 70.67 | 70.64 | 0.03 | 67.13 | **3.54** |
| C-5′′′ | 68.73 | 68.68 | 0.05 | 68.68 | 0.05 |
| C-6′′ | 67.61 | 67.65 | −0.04 | 67.63 | −0.02 |
| C-B′ | 60.05 | 62.85 | −**2.8** | 62.85 | **−2.8** |
| C-B′ | 60.00 | 60.00 | 0 | 60.01 | −0.01 |
| C-B | 59.79 | 59.74 | 0.05 | 62.57 | **−2.78** |
| C-6′′′ | 18.14 | 18.13 | 0.01 | 18.13 | 0.01 |
| C=O |  | 174.16 |  | 174.07×2 |  |
| CH_3_CH_2_CH_2_- |  | 27.24 |  | 27.23×2 |  |
| CH_3_- |  | 9.41 |  | 9.39 |  |

**Figure legends**

**Figure S1.** The HPLC chromatograms of before reaction (a) and after reaction by *P. aeruginosa* cells (b) and *P. stutzeri* cells(c) catalysis

**Figure S2.** The mass spectrum of troxerutin, TME, and TDE ^*^. ^*^ Troxerutin, ESI-MS (m/z): 765.2206 (M + Na)^+^; TME, ESI-MS (m/z): 821.2483 (M + Na)^+^; TDE, ESI-MS (m/z): 877.2733 (M + Na)^+^.

**Figure S3.** The FT-IR spectra of troxerutin (a) , TME (b), and TDE (c)

**Figure S4.** The ^13^C-NMR spectrum of troxerutin (a), TME (b), and TDE (c)

**Figure S5.** The activity of alkaline phosphatase (ALP) in Caco-2 cells





**Figure S1**


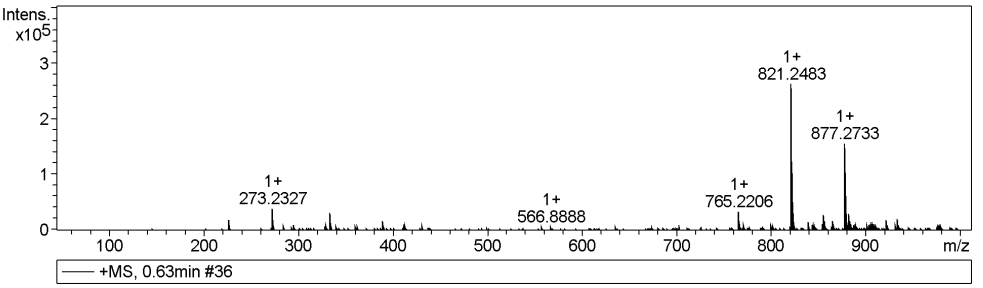


**Figure S2**





**Figure S3**


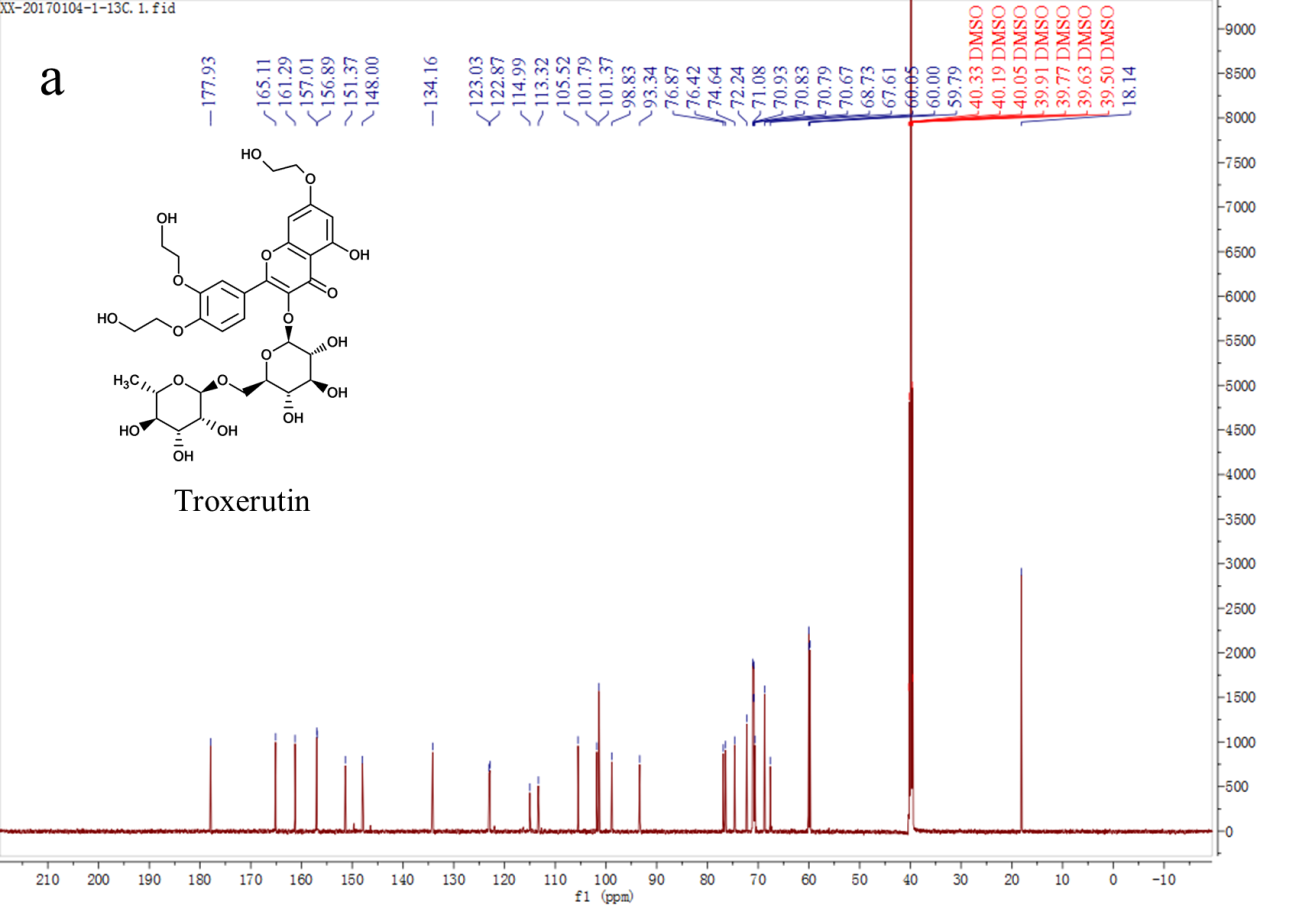

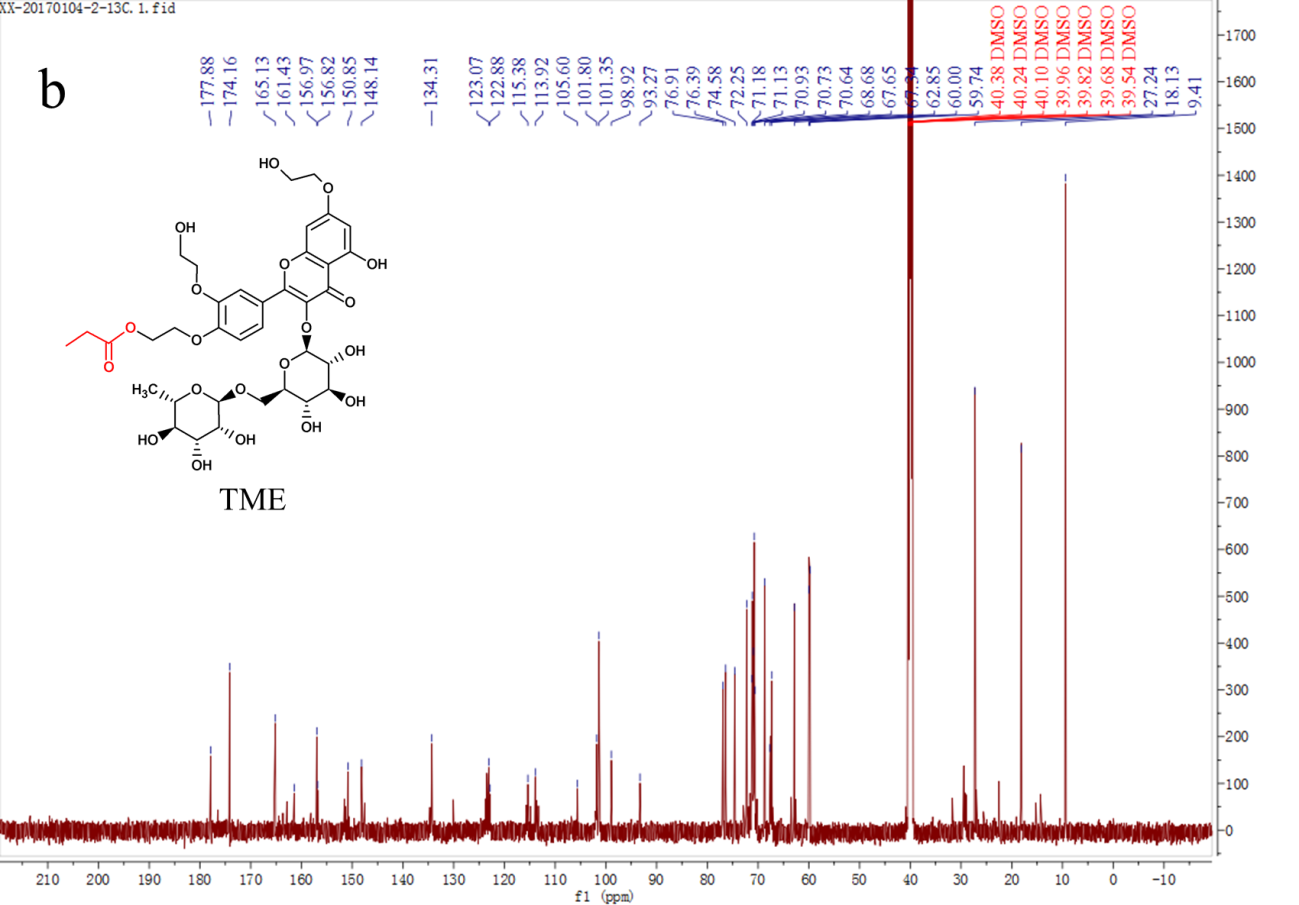

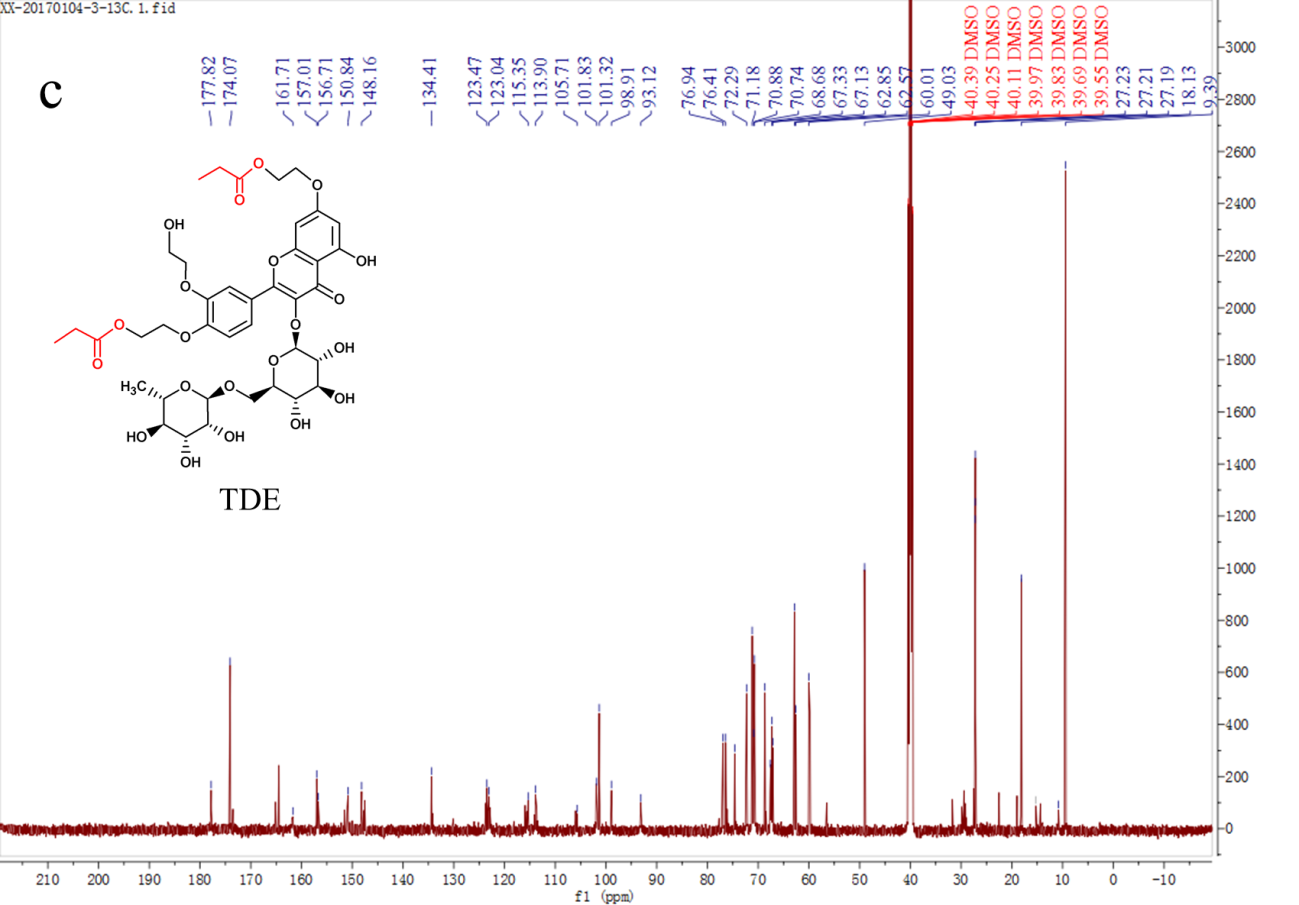


**Figure S4**





**Figure S5**
